# Complementary sample preparation strategies (PVD/BEXP) combining with multifunctional SPM for the characterizations of battery interfacial properties

**DOI:** 10.1016/j.mex.2021.101250

**Published:** 2021-01-26

**Authors:** Handian Pan, Yue Chen, Wenhui Pang, Hao Sun, Jiaxin Li, Yingbin Lin, Oleg Kolosov, Zhigao Huang

**Affiliations:** a College of Physics and Energy, Fujian Provincial Key Laboratory of Quantum Manipulation and New Energy Materials*,* Fujian Normal University, Fuzhou 350117*,* China; b Physics Department and Materials Science Institute, Lancaster University, LA1 4YB, UK; c Bruker Nano Surfaces and Metrology Division, Bruker (Beijing) Scientific Technology Co. Ltd., Beijing 100081, China; d Fujian Provincial Engineering Technical Research Centre of Solar-Energy Conversion and Stored Energy, Fuzhou 350117, China; e Fujian Provincial Collaborative Innovation Centre for Advanced High-Field Superconducting Materials and Engineering, Fuzhou 350117*,* China

**Keywords:** Thin film electrodes, BEXP, *In situ* SPM, Post-mortem, Solid-liquid interface, Lithium/sodium ion battery

## Abstract

The cathode/anode-electrolyte interfaces in lithium/sodium ion batteries act as the “gate” for the ion exchange between the solid electrode and liquid electrolyte. Understanding the interfacial properties of these solid-liquid interfaces is essential for better design high-performance lithium/sodium ion batteries. Here, we provide a novel method for studying solid-liquid interfacial properties of battery materials through combining physical vapor deposition (PVD) and beam-exit cross-sectional polishing (BEXP) followed by controlled environment multifunctional Scanning Probe Microscope (SPM). In this method, commercial battery materials can be either directly grown on the current collector substrates, or polished by obliqued Ar-ion beams to get a nanoscale flat surface which allows the multifunctional SPM to study sample directly in the liquid electrolyte or in protective oxygen/H_2_O free environment. This approach allows to investigate wide range of interfacial properties, including surface morphology, internal cracks, mechanical properties, electronic/ionic conductivity and surface potential, with nanoscale resolution in-operando during the battery cycles as well as post-mortem.

• PVD and novel BEXP methods were introduced to prepare battery powder materials as perfect specimens for nanoscale SPM characterization.
• Various physical/chemical properties of battery materials can be probed on the as-prepared specimens under liquid electrolyte using in situ/operando SPM techniques.
• Ex situ/post-mortem analyses based on the controlled environment multifunction SPM characterizations can be achieved in the BEXP polished degradation battery electrodes.

## Graphical abstract

**Figure:**
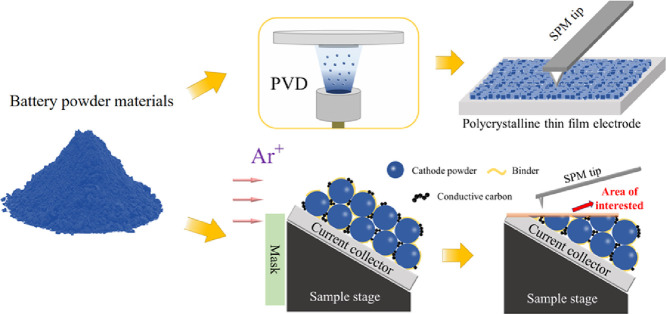

Specifications table

**Table:** 

Subject Area:	Energy
More specific subject area:	*Lithium/sodium ion Battery*
Method name:	*Sample preparation methods of battery powder materials for SPM based in situ/ex situ characterizations*
Name and reference of original method:	*None*
Resource availability:	*Beam-Exit Cross-Sectional Polishing:* https://www.lancastermaterialanalysis.co.uk/beam-exit-cross-sectional-polishing *SPM devices, in situ EC-cell and tips:* https://www.bruker.com/products/surface-and-dimensional-analysis/atomic-force-microscopes/dimension-icon/overview.html

## Method details

### Overview

Although *in situ* SPM techniques have been used as a powerful tool for battery interfacial property characterization for many years, most previous researches merely employed electrochemical Atomic Force Microscope (EC-AFM) for *operando* nano-morphology observation [1], [2], [3]. EC-AFM works well in the anode materials because the anode materials usually show visible surface morphology changes caused by the formation of solid electrolyte interphase (SEI) [4]. However, on the cathode side, the SEI (also called cathode electrolyte interphase, CEI) can only be observed after long-cycle degradation or when the cathode electrodes were charged at a relatively high voltage range [3]. Even in that case, the ultrathin cathode SEI layer is still difficult to be detected by the AFM topography mapping alone. Therefore, other surface properties, such as electronic/ionic conductivity, mechanical properties and surface potential of the electrode-electrolyte interface, should be further characterized by the *in situ* SPM for the correlation of the battery performance with its interfacial properties. To date, a range of advanced SPM techniques have been developed to operate in the liquid environment, which simultaneously enable the morphology, nanomechanical and nanoelectrical characterizations [5,6]. However, in order to obtain reliable and robust SPM measurements, these techniques generally require to be performed on idealized “model” SPM samples, such as highly oriented pyrolithic graphite (HOPG) with ultra-flat surface [2].

While such model SPM specimens can dramatically increase the characterization efficiency and image quality, improve test stability and reproducibility, the real battery electrode materials generally present more complex micro/nano particle morphology. As a result, it is difficult to obtain high resolution SPM images on micro/nano-size particles, especially in the liquid environment. In many cases, the interaction force of SPM tip and/or surface tension of liquid electrolyte could cause the detachment of particles from substrates. Many solutions, including substrate embedded single-particle (SEP) method [1], mechanical press embedded (MPE) method [3], electrochemical deposition [7], and epoxy resin embedded (ERE) method [8], have been introduced to overcome these difficulties, especially for the studies in a liquid electrolyte. However, electrochemical deposition and SEP method may introduce sample's surface contamination, and the MPE method could destroy the material microstructures and sample-substrate conductance pathway. Moreover, without the sample surface polishing process [9,10], specimens prepared by ERE method show a rough surface topography, which could cause the topography convolution during the material property measurements. Hence, in our method, we introduce two complementary strategies, physical vapor deposition (PVD) [6,^11^,12] and beam-exit cross-sectional polishing (BEXP) [13,14] methods, to prepare binder free and nano-polished/cross-sectioned composite electrode, respectively. Combination of these methods can provide efficient solution resolving long-outstanding problem for SPM studies of battery materials.

PVD method, including pulsed laser deposition (PLD), thermal/E beam evaporation and sputtering, are suitable for the deposition of metal oxide, such as Li_4_Ti_5_O_12_ [15], LiCoO_2_ [16] and LiNiMnCoO_2_ [17], which are the most commonly used battery materials. These deposition methods can directly prepare the battery materials in a thin film form on current collectors with a close to atomically flat surface. The as-prepared samples are conductive additives- and binder-free electrodes, and therefore they can be used as the perfect model for the studies of cathode/anode initial electrochemical processes by eliminating the side effects of binder and conductive additives. Without the interference signal from other components in the battery electrode, the measurement results can be directly related to the intrinsic physico-chemical properties of battery materials, and therefore allow to understand the fundamentals behind battery performance.

The post-mortem SPM conducted in a controlled environment is also becoming a powerful technique for battery degradation research [18]. For the commercial composite electrodes, during the repeated ion intercalation/deintercalation or after the long charge/discharge cycles, the insights into the internal structure and components evolutions is vitally important for better understanding their capacity fading mechanisms [19,20]. BEXP [9,10] is introduced in this paper for a first time as a useful sample preparation technique for the characterization of composite battery electrodes using multifunctional SPM. Different from conventional Ar-ion polishing method [21], a negative tilt angle was introduced in the BEXP electrode polishing process, with beam impinging on the side of the sample. This approach generated an adjustable smooth slope section consisting of battery active materials, binder and conductive additives, leaving the top surface completely intact. The dimension of the polishing area by BEXP is at millimeter scale which is much higher than Focused Ion Beam polishing. Moreover, BEXP method uses non-reactive Ar species rather than Ga^+^, minimally affecting physical and chemical properties of the material section. It should be noted that composite battery electrodes prepared by BEXP are not only suitable for *ex-situ/post-mortem* SPM characterization, but also suitable for detecting the actual failure sites in composite electrodes through *in situ/operando measurements*. With minimal topographic contrast of the section, it is essential that complementary material sensitive contrast could be used for mapping the evolutions of local nanomechanical properties [22], work function [23] and local conductivity [24] of battery materials in *operando* and *post-mortem* conditions.

Here, we introduce the complementary methods, PVD and BEXP, combining with multifunctional SPM to study physio-chemical properties on the battery electrode-electrolyte interface. The preparation of SPM specimens, assibilation/structures of *in situ* EC-cell and demonstrations of different SPM measurements were provided in this paper.

### Preparation of the specimens and in situ EC-cell kits

**Fig. 1:**
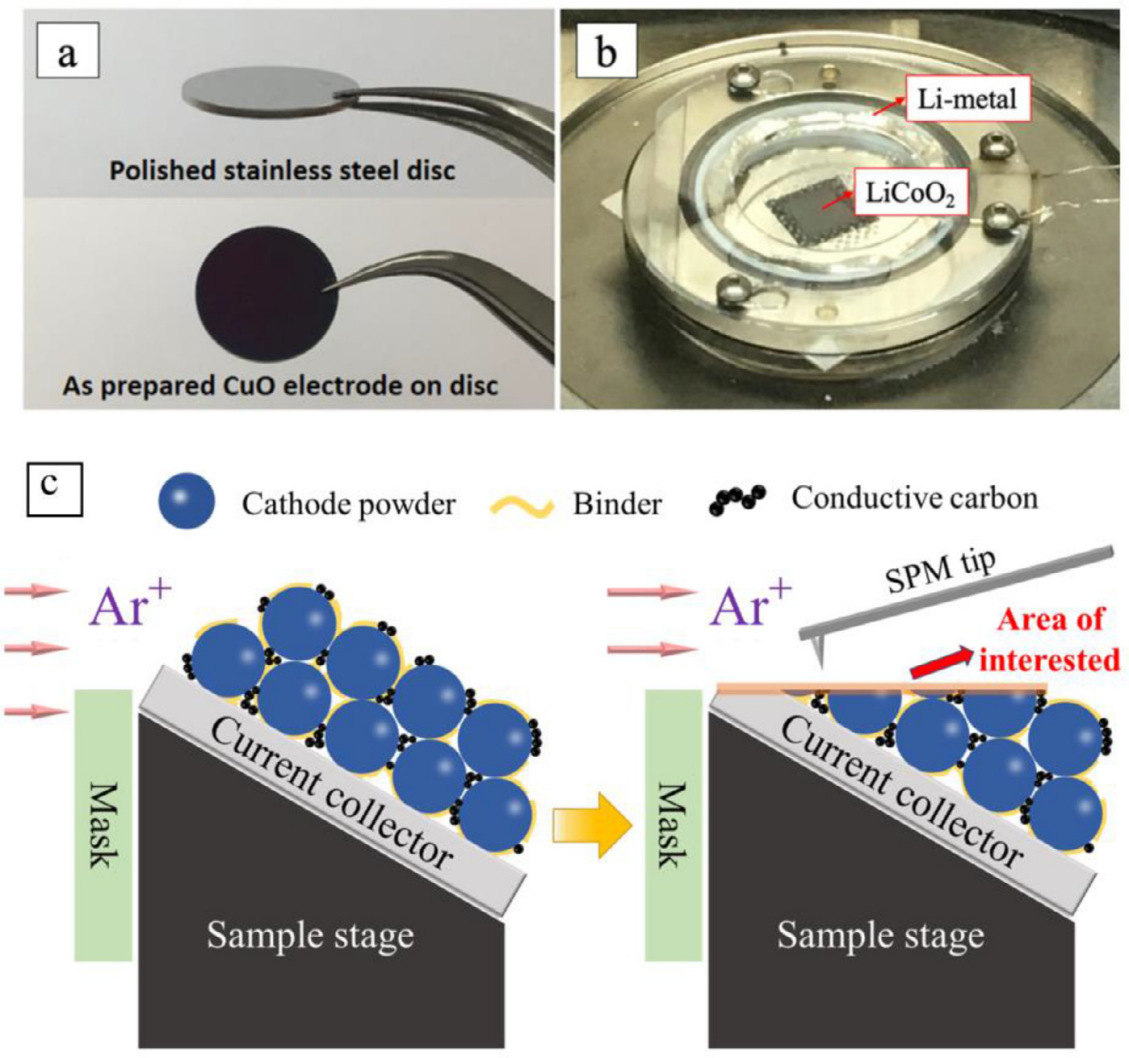
(a) The CuO anode deposited on the stainless-steel substrate [12]. (adapted from Yue et al., Journal of Solid State Electrochemistry. 23 (2019) 367-377) (b) The LiCoO_2_ cathode deposited on an alumina substrate for *in situ* SPM characterization. (c) Composite electrodes cut by BEXP method.

Fig. 1 (a) shows an example of PVD deposited CuO anode on the polished stainless-steel substrate [12]. Typically, the copper substrate should be chosen as the current collector for anodes, and aluminum should be selected for LiCoO_2_ cathode (as shown in Fig. 1b). Here, the polished stainless-steel substrate was chosen here because it is electrochemical inert when serving as the battery current collector for both cathode and anode. Moreover, the lack of the surface oxidation layer on the stainless-steel substrate surface also reduces the interfacial electronic resistance between the deposited materials and the current collector. The other advantage of PVD method is that most battery powder materials can be prepared as a thin film formed on the current collector foil through the conventional target preparation and deposition procedures.

The other complementary method, BEXP, is directly suitable for preparing composite electrodes for SPM studies. Fig. 1 (c) shows the diagram of a composite electrode cut by BEXP method that is different from the conventional cross-section polishing method. The tilt angle between the electrode and Ar-ion beam allows the cutting area to be an extended low angle slope rather than a steep cross-section that is adjacent to the undisturbed intact surface of the electrode. This makes the area of interest dramatically increased, and thereby the obtained electrode section that can be horizontally mounted on the conductive substrate for simultaneous electrochemistry and *in situ* SPM measurements. Additionally, the Ar ion polished surface does not have any contamination, which is essential for the SPM based interfacial property analyses. The detail description of BEXP method and its applications can be found in references 9 and 10.

**Fig. 2:**
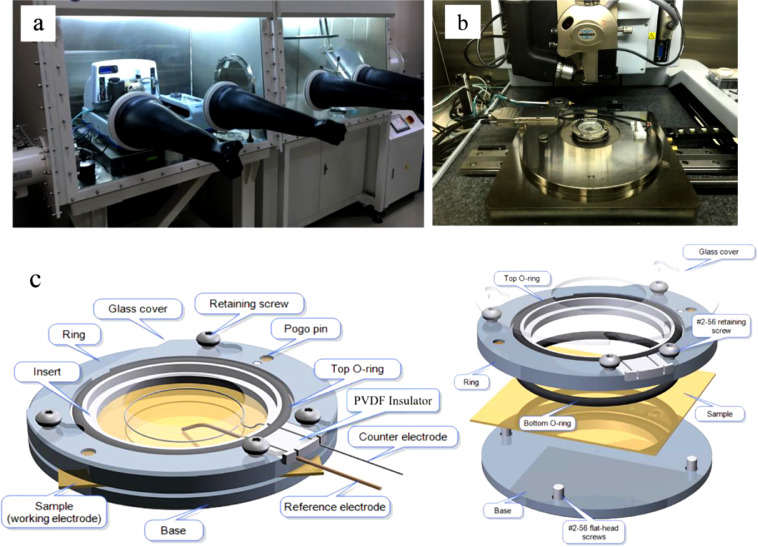
(a) and (b) The pictures of controlled environment Bruker Icon SPM. (c) The schematic diagram (left) and exploded view (right) of the *in situ* EC-cell.

The controlled environment SPM measurements are carried based on the Bruker Icon system in Ar environment protected glove box, as shown in Fig. 2 (a) and (b). The *in situ* SPM, equipped with a potentiostat-controlled electrochemical cell, can reveal the morphology, double layer thickness and electronic/ionic conductivity evolutions in the liquid electrolyte environment. Additionally, other customized advanced SPM, such as Kelvin Probe Force Microscopy (KPFM), Dielectric Electrostatic Force Microscopy (D-EFM) and Scanning Spreading Resistance Microscopy (SSRM), can be also performed in the Ar atmosphere in this system. In a nutshell, both the *in situ* (in the liquid electrolyte) and post-mortem (in Ar atmosphere) SPM measurements can be realized in such controlled-environment multifunctional SPM. The *in situ* SPM measurement can be carried out in the special designed EC-cell with a temperature controller from Bruker Company. Fig. 2 (c) presents the schematic diagram and exploded view of the *in situ* EC-cell. The materials and functions of each part are listed below:

(1) Base: The nickel-plated tungsten alloy. Four flat-head screws to secure the base to the ring (body).
(2) Ring (body): A 2205 Stainless Steel alloy.
(3) Insert: Teflon® (PTFE). An insulating insert is used to electrically and chemically isolate the electrolyte from the EC cell body.
(4) Bottom and top O-ring: Kalrez®. Used to provide a seal during the electrolyte injection.
(5) “Glass” cover: A laser-cut fused silica cover. It is used to control electrolyte evaporation during the measurements.
(6) PVDF insulator: Used to insulate the electrodes from the conductive body.
(7) Gold-plated pogo pins: Used to connect the top of the sample to the cell base.
(8) Electrodes: Working electrode (WE), reference electrode (RE, Ag/AgCl, Lithium or Sodium metal), counter electrode (CE, Platinum, Lithium or Sodium metal).

**Fig. 3:**
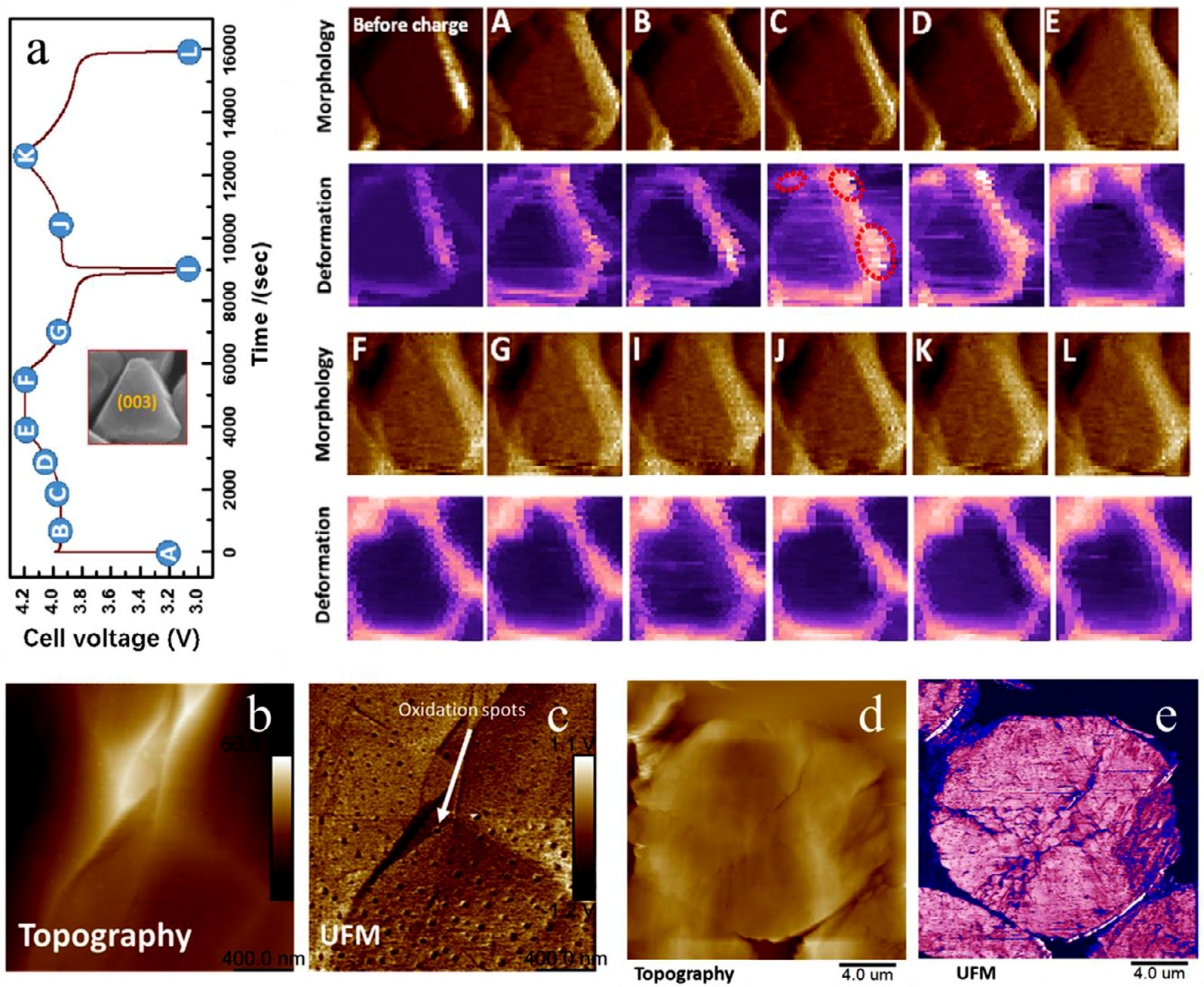
(a) The charge/discharge curves of the LiCoO_2_ (inset is the SEM image of a single crystal) thin-film electrode and the morphology and nanomechanics measurements on the single cathode crystal facet. (adapted from Yue et al., Chemical Engineering Journal. 399 (2020)) (b) and (c) Surface topography and mechanical mapping of oxidized surface of sodium cathode material exposed in the air after one week. (d) and (e) Surface topography and nanomechanical UFM mapping on the commercial nickel-rich cathode electrode cut by BEXP.

*In situ* electrochemical cell is assembled in a glove box with the contents of moisture and oxygen less than 0.1 ppm:

(1) Firstly, place the WE under investigation on the top of the nickel-plated tungsten cell base with electric contacted and fix it between the base and ring by four flat-head screws;
(2) Then, insert the PVDF insulator inside the cell body, and put CE/RE strips on the top of the insulator without contacting with WE and cell body (as shown in Fig. 1 (b));
(3) After the “glass” cover is fixed on the top of the cell through the retaining screws, the EC-cell assembly is completed;
(4) At last, a suitable amount of electrolyte is added through the hole of the top glass cover to ensure the WE, RE and CE are completely saturated with the electrolyte, so that cell is fully prepared for the measurements.

## Method validation

### Morphology and mechanical properties

The solid-electrolyte interphase layer, which governs key battery electrochemical processes, has always been the most elusive component in lithium/sodium ion batteries. So far, the *in situ/operando* SPM is the only technique that can directly observe the SEI formation and mechanical properties in liquid electrolyte inside the functioning battery. However, it should be noted that the study of the SEI formation is usually hindered by the roughness of the sample surface and complex decomposition components of battery electrodes and electrolyte. As a result, most previous studies merely carried out the SEI measurements on the HOPG surface which has atomically flat surface forming the known organic/inorganic decomposition [2,^25^,26]. Thin-film electrodes prepared by PVD and composite electrodes polished by BEXP can both provide the flat surface (nanoscale roughness), which can serve as the ideal specimens for SEI studies by SPM.

Fig. 3 shows the examples of nanomechanical property measurements on the PVD and BEXP prepared specimens by Peak force QNM (PF-QNM) [27] and Ultrasonic Force Microscopy (UFM) [28,29]. PF-QNM and UFM are advanced SPM techniques that enable the measurement of the material properties such as adhesion, elastic modulus, deformation under stress, revealing near-surface and sub-surface defects [30]. Importantly, both techniques can work under liquid electrolyte enabling the *in situ* observations in real space with nanoscale spatial resolution. As shown in the example in Fig. 3 (a), during the first two charging/discharging cycles, the evolution of morphology and surface deformation on a single triangle crystal in the thin film electrode were observed [11]. The morphology channel did not show any visible changes during the cycles. However, the deformation mapping, which represents the sample surface deformation at the peak force point, and hence inversely proportional to the elastic moduli of the material, shows larger values at the boundary region around the grain top facet. The high deformation region can be attributed to the local region covered by soft organic decompositions. In other words, this result indicates that the PF-QNM measurement can directly distinguish the ultrathin SEI layers formed on the thin-film cathode surface in the liquid electrolyte.

It should be pointed out that the PF-QNM measurement requires that the tip is mechanically stiff enough to generate the sample surface deformation. Thus, PF-QNM is well suitable for detecting the soft organic components (topmost SEI layer and adsorption molecules) formed on the surface of battery materials, but it is not appropriate for the studying of surface components which have high Young's module values, such as the inorganic oxidation products (NaOH or Na_2_CO_2_) in sodium ion battery cathode [31]. Other mechanical measurements such as Contact Resonance Atomic Force Microscopy (CR-AFM) and UFM can make up for this drawback [32,33]. As shown in Fig. 3 (b) and (c), the UFM channel can detect the surface oxidation nano-sports formed on the sodium cathode electrode surface, which provides an effective characterization method for studying the air stability of sodium cathode materials. Another advantage of UFM is its capability to detect the mechanical defects underneath the sample surface. In layered cathode materials, electrochemical de-lithiation induced phase transition usually couples with mechanical effects leading to its performance degradation [34]. The real-time observations of cracks formation and quantitative measurements of these would provide very valuable information. As shown in Fig. 3 (d) and (e), combining UFM and BEXP methods, the captured UFM images of the composite electrode cut by BEXP can not only differentiate the different areas with distinct mechanical stiffnesses (active cathode material, polymer binder and carbon conductive additives), but also detect the cracks inside the polished cathode particles. The dynamic formation of internal cracks can be further observed in this BEXP polished specimen through using *in situ* EC-UFM. Overall, combination of PVD and BEXP show a promising application ability for advanced SPM based nano-mechanical measurements.

### Electronic/ionic transportation properties

**Fig. 4:**
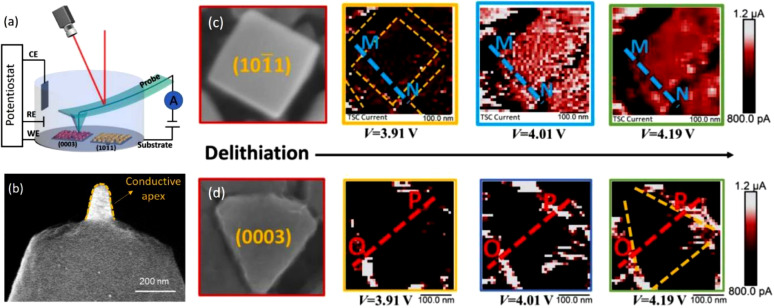
(a) Schematic of AFM-based conductivity measurements in the liquid electrolyte. (b) SEM image revealing exposed Pt-coated tip apex with ~50 nm end tip diameter and ~200 nm tip height (adapted from Nellist et al., Nanotechnology. 28 (2017) 095711). (c) and (d) *in situ* conductivity measurements on different crystal facets of LiCoO_2_ cathode. (adapted from Yue et al., Chemical Engineering Journal. 399 (2020)).

Understanding the electronic/ionic transportation properties in battery materials is vitally important for improving battery high-rate performance. AFM based conductivity measurements are powerful techniques for nanometer-scale electrical characterization on a wide range of battery materials. Traditionally, these measurements have been categorized into two classes: Conductive AFM (CAFM), which covers the higher current range (sub-nA up to μA), and Tunneling AFM (TUNA), which covers the lower current range (sub-pA up to nA). As shown in Fig. 4(a), because of practical limitations, most *in situ* conductive AFM measurements have been restricted to the Contact Mode with a voltage biased conductive tip operating in a liquid electrolyte. As a result, there are two key factors for measuring the electronic/ionic transportation properties in the liquid ionically conductive environment: 1) vertical (along the tip-sample direction) homogeneity of the specimen; 2) stray current of the tip. The vertical homogeneity of specimen relates to the uniformity of specimens. The specimen prepared by PVD on the current collector surface shows uniform specimen thickness and pure sample composite, which is vital for the comparison of current signal difference derived from the material intrinsic transportation properties. As for the stray capacitance of the tip and stray current associated with electrochemical reactions from chemical impurities, these can be significantly reduced by using Bruker SECM tip [35] (as shown in Fig. 4b) due to its small electrically exposed apex. Taking the advantages of PVD prepared thin-film samples and the elaborately designed SECM tip, the measurements of conductivity evolutions on the different crystal facets of LiCoO_2_ cathode are presented in Fig. 4 (c) and (d). As shown in the figures, the CAFM current on both grains was increasing at the beginning of the charge (de-lithiation) due to the insulator to metal transition, however, reduced at the high voltage limit due to the formation of electronic insulative SEI layers. Therefore, this SPM current measurements reveal the nanoscale electronic/ionic transportation properties and underline mechanism of SEI formation in a charging battery, providing new insights for understanding the conductivity polarization and CEI formation of cathode electrodes [11]. Similarly, the degradation studies by using post-mortem SSRM on Ar ion polished composite electrodes were also reported in Ref. [18], which could also be realized by using BEXP +SSRM.

### Ex situ intrinsic electric properties

**Fig. 5:**
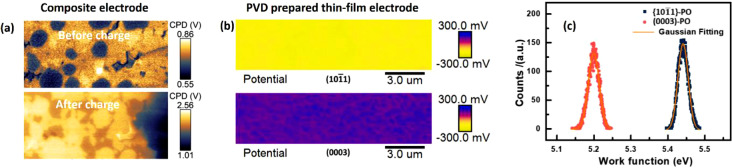
(a) CPD images of the composite electrode before and after charge. (adapted from Masuda et al., Nanoscale. 9 (2017) 893-898). (b) and (c) CPD images and converted work function distribution functions of the cathode thin-film electrode. (adapted from Yue et al., Chemical Engineering Journal. 399 (2020)).

Controlled environment KPFM, together with conductive AFM, has been recognized as the two most widely used complementary nanoscale electrical characterization tools for battery researches. As shown in Fig. 5 (a), Hideki et al. performed the KPFM on the Ar-ion polished composite electrode inside the N_2_ flow glove box, the change in potential distribution arising from battery charging was directly observed in the polished cross-section [36]. Alternatively, to study the intrinsic surface potential of active materials, we can also perform the KPFM measurements on the surface of the thin-film electrodes prepared by sputtering [11]. As shown in Fig. 5 (b), the uniform surface potential distribution over the whole scanning region was obtained on these thin film electrodes prepared by PVD. Moreover, in Fig. 5 (c), the work function distribution functions converted from the obtained surface potential map have a small FWHM value of about 50 meV. The precise determination of surface electron level can provide the reliable evidence of charge transfer between the electrolyte decomposition products and battery materials.

## Conclusion

To sum up, the PVD and BEXP methods can prepare model electrode with an atomic-flat surface without contaminations. The as-prepared model electrode can be directly used as the specimen for multifunctional SPM measurements, to perform mechanical, electrical conductivity and other interfacial property characterizations which require sample tip contact interactions during the measurements. Moreover, by performing the multifunctional SPM under a controlled environment, the initial electrochemical process of the electrode-electrolyte interface can be well detected both through *in situ/operando* and *ex situ* SPM measurements in these model electrodes.

## Declaration of Competing Interest

The authors declare that they have no known competing financial interests or personal relationships that could have appeared to influence the work reported in this paper.
